# Atypical clinical and radiological presentations of lumbar spinal tuberculosis mimicking a spinal tumor: A case report

**DOI:** 10.1097/MD.0000000000032730

**Published:** 2023-01-20

**Authors:** Kodai Miyamoto, Hiroki Imada, Shinsuke Yoshida, Hideaki Oka, Shunpei Iida, Kazuo Saita, Satoshi Ogihara

**Affiliations:** a Department of Orthopaedic Surgery, Saitama Medical Center, Saitama Medical University, Saitama, Japan; b Department of Pathology, Saitama Medical Center, Saitama Medical University, Kawagoe, Saitama, Japan; c Department of Neurosurgery, Saitama Medical Center, Saitama Medical University, Saitama, Japan; d Department of General Medicine, Saitama Medical Center, Saitama Medical University, Saitama, Japan.

**Keywords:** diagnosis, infection, lumbar spine, spinal tuberculosis (TB), spinal tumor

## Abstract

Tuberculosis (TB) is an important infectious disease worldwide. Classical spinal TB has specific radiological findings involving adjacent vertebral bodies with destruction of the intervertebral disc and involvement of paravertebral soft tissues with cold abscess formation. However, a few cases not exhibiting the radiological characteristics of TB spondylitis have been reported. We report a rare case of lumbar spinal TB with atypical clinical and radiological presentations that was difficult to differentiate from a malignant spinal tumor.

**Patient concerns:**

A 21-year-old man, who had immigrated to Japan from the Philippines 5 years ago, without a significant medical history, presented with back pain lasting 1 month and progression of gait disturbance 2 weeks prior to presentation.

**Diagnosis:**

Laboratory tests showed normal blood cell counts and normal value of C-reactive protein levels. Preoperative imaging studies indicated a possible spinal tumor. However, histopathologic findings of the epidural soft tissues at the first surgery led to the diagnosis of spinal mycobacterial infection. The diagnosis of spinal TB was confirmed by a positive culture of *Mycobacterium tuberculosis* obtained at the second surgery.

**Interventions:**

Given the progressive nature of neurologic deterioration, instead of needle biopsy, we proceeded with surgical intervention 8 days after admission; simultaneous neural decompression and open biopsy. Histological findings of the excised epidural soft tissues led to the diagnosis of spinal mycobacterial infection. We performed the second surgery involving additional resection of epidural soft tissues for further dural decompression and to obtain specimens for mycobacterial culture. Immediately after the second surgery, the patient commenced combination therapy with anti-tuberculous drugs.

**Outcomes:**

The patient demonstrated significant recovery of motor function in the lower extremities, and was able to run at 2 months after the second surgery. The epidural granulomas completely disappeared on magnetic resonance imaging 3 months postoperatively.

**Conclusion:**

Atypical clinical and radiological presentations of spinal TB present a challenge for appropriate diagnosis and early treatment. Even in developed countries where there are very few spinal TB patients, clinicians should be aware that spinal TB is an important differential diagnosis, especially in elderly patients or patients coming from countries with a middle-high prevalence of TB.

## 1. Introduction

Tuberculosis (TB) is an important infectious disease worldwide. Specific radiological findings of classical spinal TB include involvement of adjacent vertebral bodies with destruction of the intervertebral disc and paravertebral soft tissues with cold abscess formation.^[1,2]^ With advanced imaging modalities and the development of appropriate chemotherapy, many cases of spinal TB are readily diagnosed and treated successfully. However, as previously reported, a small number of spinal TB cases with atypical radiological presentations can be a diagnostic dilemma for surgeons.^[3]^ Here, we report an extremely rare case of lumbar spinal TB, with atypical clinical and radiological presentations, that was difficult to differentiate from a malignant spinal tumor.

## 2. Case report

**Figure 1.**
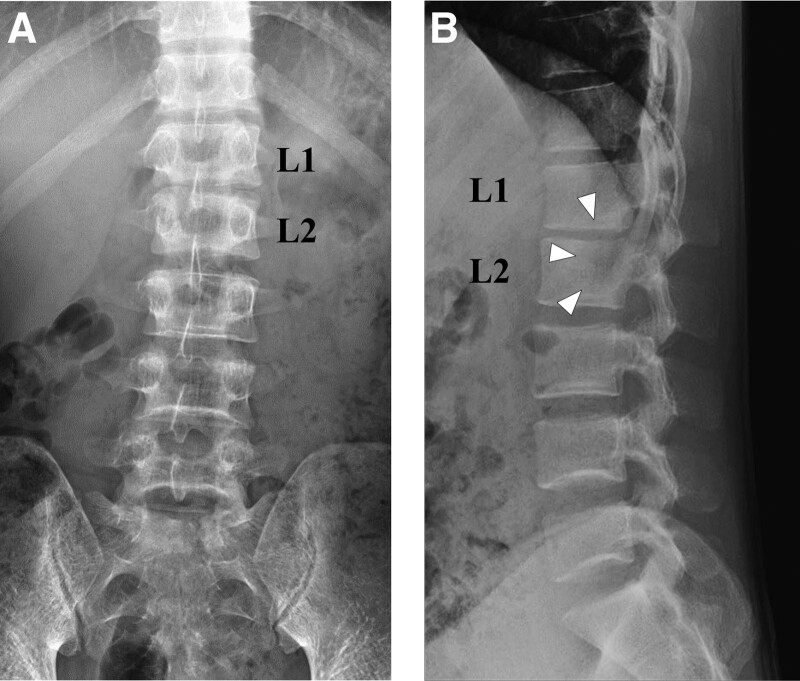
(A) Preoperative anterior–posterior view of plain radiograph showing a mild reduction in the L1–L2 intervertebral disc height. (B) Preoperative lateral view of plain radiograph showing mild osteolytic findings on the posterior side of the L2 vertebra (arrowheads).

**Figure 2.**
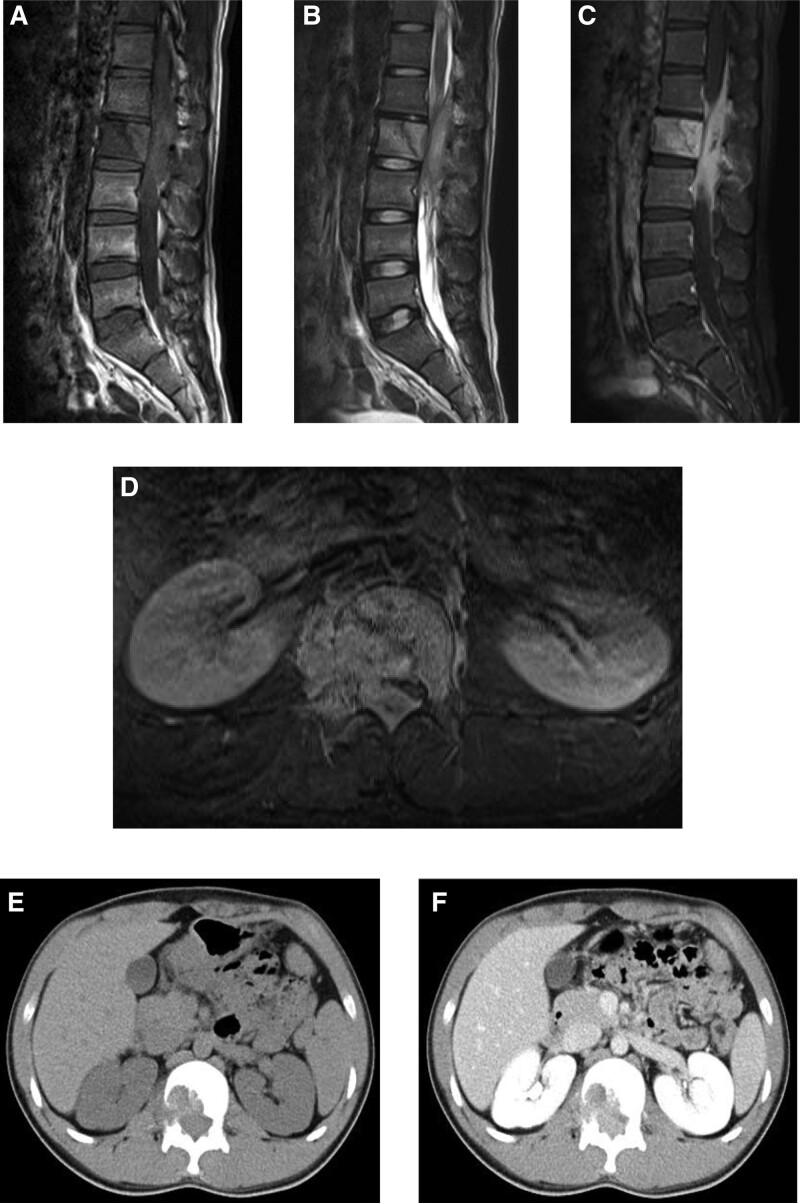
(A) Preoperative sagittal T1-weighted magnetic resonance (MR) image showing that the L2 vertebral body and contiguous epidural space-occupying lesion are of moderately heterogeneous iso signal intensity. (B) Preoperative sagittal T2-weighted MR images showing that the L2 vertebral body and contiguous epidural space-occupying lesion have moderately heterogeneous high signal intensity. (C) Preoperative sagittal gadolinium-enhanced fat-suppressed MR image showing that the L2 vertebral body and contiguous epidural space-occupying lesion have a high signal intensity. (D) Preoperative axial gadolinium-enhanced fat-suppressed MR axial image demonstrating that the lesion extended from the L2 vertebral body to the right extravertebral and epidural spaces, severely compressing the dural tube from the right side. (E) Preoperative axial computed tomography (CT) image showing an osteolytic lesion on the right posterior side of the L2 vertebral body. (F) Preoperative axial CT with contrast enhancement showing that the space-occupying epidural lesion was moderately enhanced.

A 21-year-old man who had immigrated to Japan from the Philippines 5 years ago, without a significant medical history, presented with a complaint of back pain lasting 1 month and progression of gait disturbance 2 weeks prior to presentation. Neurological examination revealed paresthesia of the lower limbs as well as weakness of the proximal and distal muscles in bilateral lower extremities (manual muscle testing grade: 3/5 on the right and 4/5 on the left); the patient was urgently hospitalized. Plain radiographs revealed mildly reduced L1 to 2 intervertebral disc height and a mild osteolytic lesion on the posterior side of the L2 vertebra (Fig. 1). Preoperative magnetic resonance imaging (MRI) demonstrated that the L2 vertebral lesion had heterogeneous iso signal intensity on T1-weighted imaging and heterogeneous high signal intensity on T2-weighted imaging. The gadolinium-enhanced view revealed that the lesion with high contrast enhancement extended from the L2 vertebral body to the epidural space and severely compressed the dural tube from the right (Fig. 2A–D). Laboratory tests showed normal blood cell counts (hemoglobin: 16.2 g/dL, white blood cell count, 6400/μL). The C-reactive protein (CRP) level was normal (0.15 mg/dL), erythrocyte sedimentation rate (ESR) was very slightly elevated (18 [normal: 0–15] mm/h), and no positive tumor markers were identified. Based on the symptoms and all examination findings, neoplasia in the lumbar spine was suspected; however, it was not clear whether it was a primary or metastatic lesion. Contrast-enhanced computed tomography (CT) of the neck, chest, spine, abdomen, and pelvis was performed to characterize and identify the primary lesion; however, no primary tumor or lesion (including the lung and lymph nodes) was found. CT revealed an osteolytic lesion in the right posterior side of the L2 vertebral body with a moderate enhancement effect (Fig. 2E and F).

Exacerbation of paralysis in his bilateral lower limbs (manual muscle testing grade of the proximal and distal muscles: 2/5 on the right and 3/5 on the left) was observed 6 days after hospitalization. Given the progressive nature of neurologic deterioration, instead of needle biopsy, we proceeded with surgical intervention 8 days after admission; this involved simultaneous neural decompression and open biopsy. We performed L1 to L3 posterior decompression involving partial removal of the epidural soft tissue mass. Intraoperative findings revealed remarkable bone fragility of the L1 to L3 lamina and reddish epidural soft tissue mass compressing the dural tube that was adhered to the underlying dural surface; the epidural soft tissue was partially resected, and the neural elements were decompressed. Fluid pooling in the epidural space was not observed.

**Figure 3.**
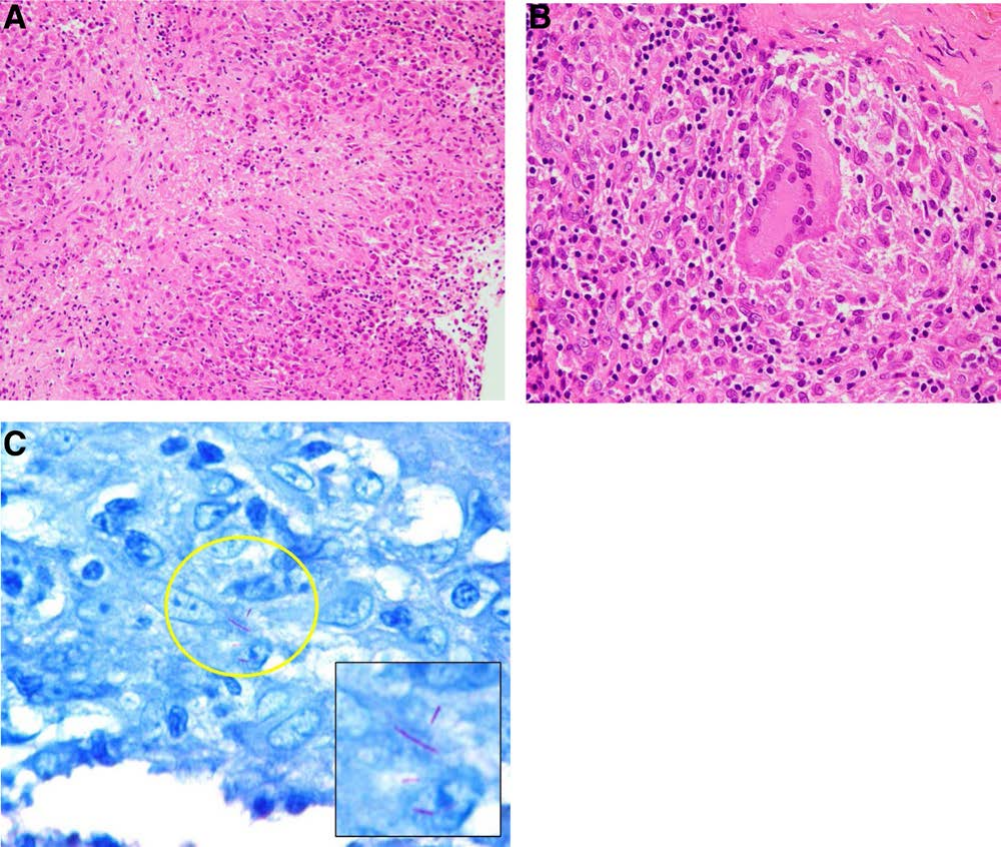
(A) Aggregates of lymphohistiocytic infiltrate with central coagulation necrosis are seen, indicative of epithelioid cell granuloma with caseous necrosis. (B) Epithelioid cell granuloma included multinucleated giant cells, some of which are horseshoe-shaped. (C) Ziehl–Neelsen staining shows a few small acid-fast bacilli, suggestive of mycobacterial infection.

Histologically, the excised epidural soft tissue demonstrated epithelioid cell granuloma with caseous necrosis. Ziehl–Neelsen stain revealed a few acid-fast bacilli (Fig. 3); therefore, mycobacterial infection was suspected. We expected a spinal tumor lesion preoperatively; therefore, bacterial culture studies were not performed initially. Results of the human immunodeficiency virus test and mycobacterial culture of sputum were negative. After acquiring the patient’s informed consent for reoperation, we performed a second surgery involving additional resection of epidural soft tissues for additional dural decompression and to collect specimens for mycobacterial culture, including drug sensitivity specimens. Immediately after the second surgery, the patient was started on combination therapy with anti-TB drugs (rifampicin [RFP], pyrazinamide, isoniazid [INH], and ethambutol [EB]) for 2 months, and then subsequently, 3 drugs (RFP, EB, and INH) for 1 month, and 2 drugs (RFP and INH) for 4 months.

**Figure 4.**
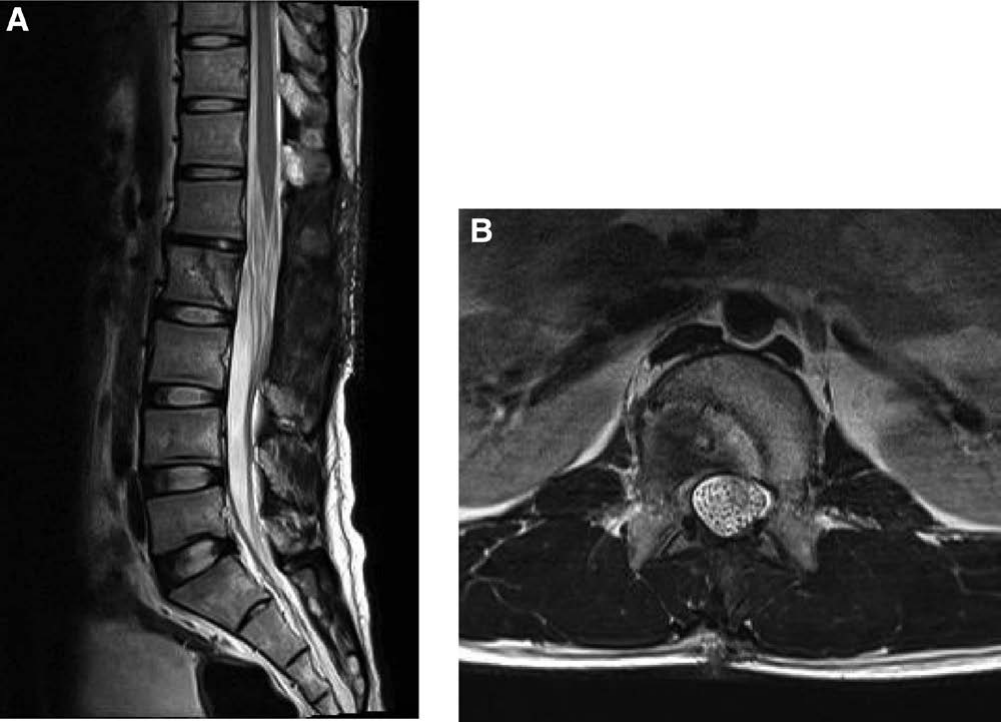
(A) Sagittal T2-weighted magnetic resonance (MR) image 3 months postoperatively, and (B) axial T2-weighted MR image 3 months postoperatively showing that epidural granulation tissues had completely disappeared, and dural tube had completely decompressed.

The diagnosis of spinal TB was confirmed by a positive culture of *Mycobacterium tuberculosis* with drug sensitivity to RFP, ISZ, EB, and pyrazinamide. The patient exhibited a marked recovery in motor function in the lower extremities. He was able to walk without aid at 1 month and run at 2 months after the second surgery. The epidural tissues disappeared completely on MRI 3 months after the second surgery (Fig. 4). Normal walking and running abilities of the patient were maintained at the final follow-up (18 months postoperatively).

The approval for this study was obtained from the institutional review board of Saitama Medical Center, Saitama Medical University (No. 1969-III). Written informed consent was obtained from the patient for publication of this case report and accompanying images.

## 3. Discussion

Advances in diagnostic imaging technologies have led to successful preoperative diagnosis of spinal TB. Typical cases of spinal TB with specific radiological findings (involvement of several contiguous vertebral segments, destruction of the intervertebral disc, large intraosseous and paraspinal cold abscesses, the presence of calcification of soft tissue shadows, and spinal kyphotic deformity) can be diagnosed easily, leading to a prompt commencement of appropriate treatment.^[4,5]^ However, in the present case, the imaging findings were highly atypical for spinal TB. No spinal abscess formation was observed on MRI and CT. Additionally, during intraoperative observations, all epidural space-occupying lesions were found to be tuberculous granulomas without liquid component. Moreover, contrast-enhanced CT of the neck, chest, abdomen, and pelvis did not reveal any major lesions of TB, which made the diagnosis of spinal TB difficult. These findings made it difficult to differentiate the present case from tumoral spinal diseases such as spinal malignant lymphoma.

Regarding laboratory blood tests, it has been reported that CRP levels and ESR are elevated in many typical cases of spinal TB and that ESR shows a relatively dominant elevation compared with CRP levels.^[6–8]^ ESR is reported to be a sensitive marker of spinal TB and can be used to monitor therapeutic response; however, its low specificity is a concern.^[7]^ Wang et al reported some cases of spinal TB where patients had normal CRP levels and ESR < 20 mm/h, although the number of cases was limited.^[9]^ In the current case, the preoperative blood test showed CRP levels to be within the normal range and only a very slight increase in ESR, further complicating the accurate diagnosis because infectious diseases could not be suspected. Taken together, our findings suggest that clinicians should be aware that, unlike purulent spondylitis caused by other bacteria, spinal TB cannot be ruled out even if the blood tests for the inflammatory markers return negative.

According to a clinical epidemiological survey, the incidence of spinal TB is the highest in developing countries including India, Indonesia, China, Nigeria, Pakistan, and South Africa.^[8,10]^ On the other hand, as the prevalence of TB is declining in developed countries, opportunities for clinicians to diagnose new patients with spinal TB are decreasing.^[7]^ A recent clinical epidemiological survey showed that many newly diagnosed TB cases in developed countries, including Japan, tend to be older or foreign-born immigrated patients.^[11]^ Additionally, an epidemiological survey in Japan revealed that the proportion of foreign-born persons is especially high among the younger patients diagnosed with TB (62.9% of those aged between 20 and 29 years).^[12]^ Therefore, in cases where atypical lesions are present in the spine, it is important for clinicians to consider differential diagnosis of spinal TB, even in developed countries, especially in older patients^[13]^ or immigrants, regardless of their age, from countries with a middle-high prevalence of TB.^[14]^

In conclusion, atypical clinical and radiological presentations of spinal TB can be a challenging in terms of appropriate diagnosis and early treatment. However, even in developed countries, clinicians should be aware that spinal TB is an important differential diagnosis, especially in older patients or patients coming from countries with a middle-high prevalence of TB.

## Acknowledgments

We are grateful to Kazutaka Izawa, MD, Department of Orthopaedic Surgery, National Hospital Organization Toneyama Hospital, for providing his expertise and sharing his clinical experiences regarding spinal tuberculosis.

## Author contributions

**Conceptualization:** Satoshi Ogihara.

**Data curation:** Hiroki Imada, Shinsuke Yoshida, Hideaki Oka, Shunpei Iida, Kazuo Saita, Satoshi Ogihara.

**Investigation:** Satoshi Ogihara.

**Writing – original draft:** Kodai Miyamoto.

**Writing – review & editing:** Satoshi Ogihara.
